# Effects of a novel nutraceutical combination (BruMeChol™) in subjects with mild hypercholesterolemia: study protocol of a randomized, double-blind, controlled trial

**DOI:** 10.1186/s13063-020-04551-4

**Published:** 2020-07-06

**Authors:** Anna Rita Bonfigli, Olga Protic, Fabiola Olivieri, Alberto Montesanto, Gelsomina Malatesta, Raffaele Di Pillo, Roberto Antonicelli

**Affiliations:** 1 Scientific Direction, IRCCS INRCA, Ancona, Italy; 2 Cardiology Unit, IRCCS INRCA, Via della Montagnola 81, 60131 Ancona, Italy; 3 Department of Clinical and Molecular Sciences, DISCLIMO, Università Politecnica delle Marche, Ancona, Italy; 4 Center of Clinical Pathology and Innovative Therapy, IRCCS INRCA, Ancona, Italy; 5 Department of Biology, Ecology and Earth Sciences, University of Calabria, Rende, Italy

**Keywords:** Dietary supplements, Hypercholesterolemia, Cardiovascular diseases, Olive oil, Phytosterols, Bergamot fruit, Vitamin K2, Randomized controlled trial

## Abstract

**Background:**

Elevated cholesterol levels and systemic inflammation are considered relevant risk factors for cardiovascular disease (CVD) development and progression. Increasing evidence suggests that cholesterol-lowering and inflammation-lowering nutraceuticals are useful in the management of moderate hypercholesterolemia. Here, we describe the study protocol of a clinical trial aimed to evaluate the cholesterol and inflammatory lowering effect of an innovative dietary supplement (BruMeChol™, Mivell S.r.l., Italy), composed of a mixture of extracts of bergamot and olive fruits in association with vitamin K2 in subjects with mild hypercholesterolemia.

**Methods:**

The study was planned as a randomized, double-blind, placebo-controlled, parallel group clinical trial for 12 weeks at the Cardiology Unit of the IRCCS INRCA of Ancona, Italy. A total of 125 subjects (age ≥ 40 years) with mild hypercholesterolemia (total serum cholesterol levels ≥ 200 and ≤ 250 mg/dl) will be recruited. Intervention arm participants will take one capsule of dietary supplement two times a day, 15 min before the main meal. Control arm participants will receive one capsule of placebo in the same way. The dietary supplement capsule contains the following ingredients: phytosterols, flavonoid-rich extract of bergamot fruit (*Citrus bergamia*), flavonoid-rich extract of olive fruit (*Olea europaea*), and vitamin K2. Participants will undergo a medical evaluation and chemical-clinical examinations, which include lipid profile, glycemia, biomarkers of renal, liver and cardiac/muscular functions, interleukins (IL 6, IL-32, IL-37, and IL-38), and innovative mediators of inflammation such as inflamma-miRs (miR-21 and miR-146a), at baseline, and after 6 and 12 weeks of treatment. The decrease in total cholesterol levels and inflammatory biomarkers will be the primary and secondary endpoints of the study.

**Discussion:**

This protocol study, planned to verify the effects of BruMeChol™ dietary supplementation in subjects with mild hypercholesterolemia, could also contribute to new study designs for next large-scale multicenter clinical trials.

**Trial registration:**

Australian New Zealand Clinical Trials Registry: ACTRN12619000170123. Retrospectively registered on 5 February 2019

## Background

Cardiovascular diseases (CVDs) in industrialized countries are the leading cause of morbidity and mortality and one of the most widespread diseases with expensive health care [1, 2]. Elevated serum cholesterol concentrations (hypercholesterolemia) are one of the main risk factors for atherosclerosis progression that can lead to myocardial infarction, stroke, and peripheral vascular disease.

Statins are the reference drug for the treatment of hypercholesterolemia. However, patients with indications for the statin therapy as primary prevention most often prefer not to take the drugs for possible side effects and attempt to reduce the total cholesterol levels in serum by improving their lifestyle. The lifestyle changes recommended are smoking cessation, no alcohol consumption, increasing physical activity, and maintaining a healthy weight [3]. When all these efforts do not give results, the intake of nutraceutical substances could be considered in improving the lipid profile.

Increasing evidence supports the beneficial effects of plant components and vitamins in maintaining the physiological levels of serum cholesterol, thus contributing to the prevention of atherosclerosis. Plant polyphenols are the starting point in developing new supplements designed to improve chronic inflammatory states, combat atherosclerosis, and the risk of thrombosis related to CVDs [4]. *Citrus bergamia*, also known as bergamot, has shown a significant degree of hypocholesterolemic as well as antioxidant/radical effects [5]. In particular, this fruit has flavanones that can act as natural statins [6]. The use of natural bergamot-derived polyphenols may allow patients undergoing statin treatment to reduce effective doses [7]. Olive tree polyphenols have properties of medical interest like anti-atherogenic, antihepatotoxic, hypoglycemic, anti-inflammatory, antitumor, antiviral, and immunomodulatory activities [8, 9]. Phytosterols are constituents of plant cell walls. Due to the structural similarity with cholesterol, when they are ingested with plant foods, they reduce cholesterol absorption from the gut. It was shown that the intake of plant sterols of 1.6–2 g a day reduces cholesterol absorption from the gut of 30%, as well as plasma low-density lipoprotein (LDL) cholesterol levels of 8–10% [10]. The role of vitamin K2 in atherosclerosis prevention is based on its ability to activate a group of proteins that keep calcium in the bones, out of the arteries [11–13]. When there is a lack of vitamin K2, these proteins cannot be activated, resulting in calcium loss [14, 15].

Starting from the knowledge of the beneficial effects of each abovementioned compound on cholesterol levels, we hypothesized that a new dietary supplement consisting of a mixture of flavonoids extracted from bergamot, olive polyphenols, plant sterols, and vitamin K2 could reduce total cholesterol levels also through their synergistic effect. Besides, it could also prevent atherosclerotic plaque formation by its antioxidant and anti-inflammatory properties.

## Methods/design

### Hypothesis

We hypothesized that the intake of this new dietary supplement, containing molecules with potential effects on lipid metabolism, could reduce after 12 weeks the total cholesterol levels in serum of subjects with mild hypercholesterolemia. We also hypothesize that this dietary supplement could decrease serum level of circulating inflammatory marker such as high-sensitive C-reactive protein (*hs*-CRP), as well as particular interleukins and specific circulating inflamma-miRs.

### Primary objective

The primary aim of the study is to evaluate the efficacy and safety of BruMeChol™ dietary supplement to reduce total cholesterol levels in serum of patients with mild hypercholesterolemia.

### Secondary objectives

The study will evaluate the change in levels of circulating inflammatory marker like hs-CRP and inflammatory cytokines such as interleukin 6 (IL6) [16], interleukin 32 (IL32) [17], interleukin 37 (IL37) [18], and interleukin 38 (IL38) [19], as well as a variation in the expression of specific circulating inflamma-miRs (miR-21 and miR-146a) [20]. Moreover, the association between cardiovascular (CV) risk score and lipidic and inflammatory parameters measured at baseline will be evaluated.

### Study design

**Fig. 1:**
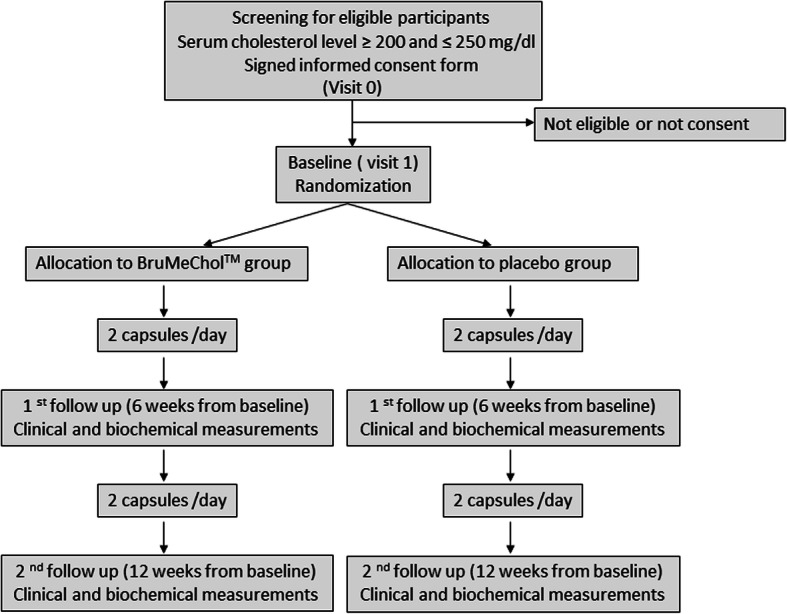
Schematic representation of study design

This interventional, randomized, double-blind, placebo-controlled clinical trial for 12 weeks will evaluate the effect of the new dietary supplement in reducing total serum cholesterol levels. The study will be conducted at the Cardiology Unit of the IRCCS INRCA of Ancona, Italy. The subjects’ involvement in the study will end after 12 weeks. The study has been retrospectively registered with the Australian New Zealand Clinical Trials Registry (ACTRN12619000170123). Figure 1 provides an overview of the study.

### Sample size and statistical considerations

In the present study, 125 subjects will be recruited, randomized according to a 1:1 ratio in the intervention and the control arm. To find a significant difference in cholesterol levels between the two treatments (BruMeChol™ vs. placebo) at the end of the 2nd follow-up, assuming a medium effect size of the proposed treatment (*d* = 0.5), we should study at least 118 subjects (at least 59 for treatment arm) with a power of 0.85 and a type I error probability of 0.05. By providing a dropout rate of 5%, a total of 125 participants must be recruited.

### Population

Subjects of 40 years or over afferent to the outpatient facilities of the Cardiology Unit for a medical check will be invited by the research staff to participate in the study. Subjects who meet the inclusion criteria and wish to participate in the trial procedure will have to sign a written informed consent. A total of 125 voluntary subjects with mild hypercholesterolemia will be enrolled. Trial staff will have the responsibility to withdraw enrolled patients at any time if they fail to meet the inclusion criteria.

### Inclusion criteria

- Both genders, age of 40 years or over.
- Total blood cholesterol levels ≥ 200 and ≤ 250 mg/dL.
- No history of clinically relevant cardiovascular events.

### Exclusion criteria

- A therapy with statins or food supplements with lipid-reducing effects.
- A therapy with anticoagulants, hypothyroidism or hyperthyroidism.
- Diabetes.
- Intestinal malabsorption.
- Acute illnesses
- Neoplastic disease or life expectancy less than 1 year.
- Statin non-related liver disease.
- Severe chronic renal failure.
- Allergy/intolerance to one or more components of dietary supplement or placebo.
- Participated in another clinical trial of intervention in the previous 3 months.
- Presence of cognitive disorders and other impediments that do not guarantee the correct adherence to the study treatments.
- There are current or presumed pregnancy and pregnancy planning.
- Incapacity, impossibility, or unavailability to sign the written consent.

### Randomization

After checking the inclusion and exclusion criteria, subjects’ randomization into the two parallel treatment arms will be carried out by the distribution of a blinded treatment kit containing test or placebo capsules. The capsules will be randomly assigned into the test and placebo groups at a 1:1 ratio according to the random number generated by SPSS v25.0 software package (SPSS Inc., Chicago, IL, USA).

### Blinding

The randomization will be performed using a code list created by the statisticians of the INRCA Biostatistical Center. The trial staff will not know the arm of allocation (double-blind study). The INRCA Hospital Pharmacy will prepare treatment kits assigning them the identification codes following the randomization lists. Only if necessary, for the safety of patients, the single kit code will be open by the investigators during the study period.

### Interventions

Patients will be randomized in two treatment groups:

1. The first group will be treated with two capsules of a new dietary supplement on a day.
2. The second group will be treated with two capsules on a day with placebo taken in the same way and timing of administration provided for the supplement.

#### Dietary supplement capsules

The new dietary supplement consists of flavonoids extracted from bergamot, olive polyphenols, plant sterols, and vitamin K2. This innovative combination of the above-described components, named BruMeChol™, will be provided by MIVELL s.r.l. (Jesi, AN, Italy). The BruMeChol™ dietary supplement has already received authorization for marketing from the Italian Ministry of Health. The constituents of the BruMeChol™ capsules are summarized in Table 1.

**Table 1:** Constituents of the dietary supplement BruMeChol™

*Active substances*	*Amount in capsule*
**Phytosterols**	**400 mg**
**Bergamot fruit**	**100 mg**
Total flavonoid	98 mg
Melitidin and Brutieridin	14.7 mg
**Olive fruit**	**20 mg**
Total polyphenols	19.6 mg
Hydroxytyrosol and derivatives	6 mg
**Vitamin K2**	**52 mcg (49.5% DRVs**^a^**)**

^a^Dietary reference values (DRVs) on a day (Reg. UE 1169/2011)

#### Placebo

The placebo capsule will contain 2% of magnesium stearate, 2% of silicon dioxide, and 96% of calcium phosphate. The placebo will be manufactured to have a similar appearance, shape, weight, taste, and color as BruMeChol™ capsule.

#### Treatments

The subjects will receive either two capsules of BruMeChol™ or placebo daily, one capsule took 15 min before lunch and one capsule 15 min before dinner.

### Study period

The study will last 12 weeks. The visits and the evaluations will be done as follows: screening, baseline-randomization (visit 1), 6 weeks from baseline (1st follow-up, visit 2), and 12 weeks from baseline (2nd follow-up, visit 3). The study schedule at each visit is presented in Table 2.

**Table 2:** Study schedule at each visit in the clinical trial

	Study schedule			
	Screening	Baseline (visit 1)	6th week (visit 2)	12th week (visit 3)
Informed consent form	X			
Recording demographic data	X			
Medical history taking		X		X
Physical examination ^a^	X	X	X	X
Glycemia	X	X	X	X
Renal function^b^	X	X	X	X
Liver function^c^	X	X	X	X
Thyrotropin-TSH	X			
Lipid profile^d^	X	X	X	X
Fool blood exam-FBE^e^		X	X	X
Leukocytes formula^f^		X	X	X
Electrolytes^g^		X	X	X
Cardiac/muscular markers^h^		X	X	X
C-reactive protein		X	X	X
Lipoprotein (a)		X	X	X
Interleukins^i^		X	X	X
Inflammatory-miRs^j^		X	X	X
ECG^k^		X		X

^a^Weight, height, waist circumference, and hip circumference

^b^Creatinine; estimated glomerular filtration rate by CKD-EPI equation

^c^Aspartate aminotransferase glutamic oxaloacetic transaminase (AST/GOT), alanine aminotransferase/ glutamic pyruvic transaminase (ALT/GPT)

^d^Total cholesterol_,_ triglyceride, low-density cholesterol (LDL), and high-density cholesterol (HDL)

^e^White blood cells (WBCs), red blood cells (RBCs), hemoglobin, hematocrit, mean cell volume (MCV), mean corpuscular hemoglobin (MCH), mean corpuscular hemoglobin concentration (MCHC), red cell distribution width (RDW), platelets

^f^Neutrophils, eosinophils, basophils, lymphocytes, monocytes

^g^Sodium, potassium, chlorine, calcium, phosphorus, magnesium

^h^Cardiac Markers Creatine Kinase (CK)/ myoglobin

^i^IL6, IL32, IL37, IL38

^j^miR-21 and miR-146a

^k^Electrocardiography

### Outcomes

#### Primary outcome

The primary outcome is the change in serum total cholesterol levels after 12 weeks of post-intervention commencement between experimental arm and placebo comparator arm.

#### Secondary outcomes

The secondary outcomes are changes after 6 and 12 weeks post-intervention commencement between experimental arm and placebo comparator arm in the following blood parameters: *hs*-CRP, triglycerides, low-density cholesterol (LDL), high-density cholesterol (HDL), lipoprotein(a), IL-6, IL32, IL37, IL38, miR-21, and miR-146a; and association at baseline between CV risk score evaluated by the SCORE risk charts (www.heartscore.org) and lipid (triglycerides, total, HDL, and LDL cholesterol) as well as inflammatory parameters (*hs*-CRP and IL-6).

### Procedures

#### Subjects recruitment

Advertisements in flyers and trial poster will be available to participants at the IRCCS INRCA hospital, Ancona, Italy.

#### Measurement tools

##### Medical history

Information about the presence or absence of the following data gained by a physician will be taken: hypertension, diabetes, hypercholesterolemia, hypertriglyceridemia, hyperlipidemia, and familiarity for cardiovascular diseases, anemia, chronic renal failure, chronic respiratory failure, dementia, liver disease, digestive system diseases, drug allergies, and surgical interventions.

##### Anthropometric measurements

Body weight will be measured by a calibrated electronic floor scale (FioMed s.r.l Fazzini, Vimodrone, Milano, Italy) to the nearest 0.1 kg. Height will be measured by an upright plastic portable Stadiometer (FioMed s.r.l Fazzini, Vimodrone, Milano, Italy) to the nearest 0.1 cm. Waist circumference will be measured at midway between the iliac crest and the lower costal margin at the end of normal an expiration using a flexible, plastic tape to the nearest 0.1 cm. Hip circumference will be measured at the widest part of the buttocks at the intertrochanteric level to the nearest 0.1 cm. All anthropometric measurements will be done by trained personnel.

##### Biochemical profile

Screening visit will include following measurements: total cholesterol, fasting glucose, liver profile (AST/GOT, ALT/GPT), kidney profile (creatinine, CKD-EPI e-GFR) and thyrotropin (TSH). Serum lipid profile: total, LDL, HDL cholesterol and triglycerides, liver profile (AST/GOT, ALT/GPT), kidney profile (creatinine, CKD-EPI e-GFR), fasting glucose, fool blood exam (FBE), leukocyte formula, electrolytes, cardiac/muscular markers (CK/myoglobin), CRP, and Lp(a) will be measured at baseline, 6 weeks, and on completion of the study (12 weeks from randomization). All items that will be measured at every visit are described in Table 2.

##### Clinical examinations

Seated blood pressure will be taken, two times with at least a 10-min rest between measurements using an Accoson mercury sphygmomanometer (M3 Intellisense Omron, Corman, Milano, Italy).

##### Electrocardiography

ECG will be recorded to verify the absence of using P8000 power by ESAOTE s.p.a, Firenze, Italy. The results of the ECG exam will be reported in the case report form (CRF) by the cardiologist.

##### Physical activity

Physical activity will be evaluated by using the International Physical Activity Questionnaire (IPAQ-SF) short form [21]. IPAQ-SF comprises a set of questionnaires that consider a time of person that spend being physically active in the last 7 days, for at least 10 min at a time. There are three kinds of physical activity: vigorous, moderate, and walking. Vigorous activities refer to hard physical effort that makes a person breathe much harder than usual. Moderate activities refer to moderate physical effort that makes a person breathe somewhat harder than usual. Walking includes any activity that one person has done for recreation, sport, exercise, or pleasure.

##### Other

Sociodemographic data will be evaluated using a tailored questionnaire, specifically designed for the study.

### Safety

At present, no adverse reactions related to the intake of this new dietary supplement are reported in the literature. However, in this study, all adverse events reported by the subjects or detected by the physician will be reported in the CRF, and all serious adverse events will be reported to the Ethics Review Committee.

### Retention

During the study, the participants will be encouraged by the physician to complete the follow-up. However, in case they still cannot complete the study, the motive for their withdrawal will be registered.

### Compliance calculation

Subjects will be asked to return the remaining capsules. The compliance will be evaluated by using the formula given below:

$$ \mathrm{Compliance}\ \left(\%\right)=\frac{\mathrm{Number}\ \mathrm{of}\ \mathrm{capsules}\ \mathrm{taken}}{\mathrm{Number}\ \mathrm{of}\ \mathrm{capsules}\ \mathrm{that}\ \mathrm{should}\ \mathrm{have}\ \mathrm{been}\ \mathrm{taken}}\times 100 $$

Compliance during the randomized period should be between 80 and 120%. Patients who will not be compliant with their treatment should again be carefully interviewed and reminded about the purpose and the conduct of the trial.

### Statistical analysis

Demographic, clinical characteristics and outcomes data will be summarized with counts and percentages for categorical variables, means (standard deviations) for normally distributed continuous variables, and medians (with interquartile ranges) for other continuous variables. Kolmogorov-Smirnov test will be used to check the normality of the related variables. Repeated measures analysis of variance (ANOVA) will be used to test whether treatment (between-subjects factor) differentially affect the mean values of cholesterol levels (within-subjects factor) over time. The same analysis will be also repeated for the inflammatory biomarkers that will be used as within-subjects’ factors. Mauchly’s test will be used to assess the assumption of sphericity, and Greenhouse-Geisser correction will be adopted for violations of this assumption. A significant level of 0.05 will be used throughout the study. Statistical analyses will be performed with IBM SPSS statistical software ver. 25.0 (IBM Corp., Armonk, NY, USA).

### Data and biological sample handling

The subject’s information collected during the trial will be depersonalized and protected by password, and access to data will be restricted to the trial staff by accordance with current Italian law. The complete final dataset will contain no identifying participant information, and its access will go only to the trial staff personnel.

Blood samples will be stored in the IRCCS INRCA secure facility at − 80 °C, with measures taken to ensure that specimens are kept under correct conditions always when it is stored. The expert staff that has been specially trained in sample storage and transportation would ensure that regularity issues are properly handled. With the approval of the principal investigator, the samples stored in the storage facility may be disposed of by the sample custodian. A sample disposal sheet will be completed and kept for further reference.

### Dissemination of study findings

The results of the above study will be published in local and international, peer-reviewed journals and presented at international conferences and clinical meetings.

### Ethical approval

The protocol (version 3_20-Dec-2017) study has been approved by the Ethics Committee of the IRCCS INRCA, Ancona, Italy, on 21 December 2017 (reference number: INRCA 17018). Before recruitment, the physician will give the participants complete information about the study and will provide them to sign the consent form, which has already been approved by the Ethics Committee. The model consent form and other related documentation to the participants are available from the corresponding author on request. Any possible protocol modification will be communicated to the ethical committee and to all relevant parties. Participants will also be informed that they can withdraw their consent to participate at any time and for any reason. If there will any harm to the participants of the trial, they will be compensated by the hospital’s insurance. The trial is retrospectively registered on 5 February 2019 under the Australian New Zealand Clinical Trials Registry (reference number: ACTRN12619000170123). The study will be conducted in compliance with the Declaration of Helsinki and the Good Clinical Practice (GCP) guidelines.

## Discussion

Our study describes the protocol of a clinical trial, designed for evaluation of the effects of a new dietary supplement on total cholesterol levels in patients with mild hypercholesterolemia. Randomized controlled trials (RCTs) are the “gold standard” of clinical trials, valued for their precision and robustness in assessing the impact of a nutraceutical or a food-based compound on specific diseases or related risk factors. RCTs are ideal tools for the investigation of causes and effects relationships between an intervention and a health outcome.

According to guidelines, patients with mild-hypercholesterolemia at low-to-moderate cardiovascular risk, and who perform regular physical activity and follow a healthy diet, may be treated with cholesterol-lowering functional foods or nutraceuticals to help achieve target LDL-c levels [22]. Moreover, even if the statins are the reference drug for the prevention of cardiac events associated with hyperlipidemia, an increasing number of patients refuse to use it for their possible collateral effects. The increasing availability of nutraceutical substances for the treatment of mild hypercholesterolemia can contribute to delay or prevent the use of statins. Previous studies have already explored the single beneficial effects of natural compounds, such as *Citrus bergamia*, olive tree polyphenols, phytosterols, and vitamin K2 on cholesterol levels and atherosclerosis process prevention [4, 8, 10–12]. Moreover, the effects of each these single substances on cardiovascular risk factors reduction have been studied in several RCT [23–26]. However, the synergistic effects of all these compounds were not previously tested. This is the first randomized controlled trial evaluating the effects of the intake of a new mixture of these active ingredients (BruMeChol™) in patients with mild hypercholesterolemia.

We have chosen as an experimental design a double-blind, placebo-controlled, parallel group clinical trial since it is the gold standard for assessing the cause–effect relationship of an innovative nutraceutical on selected biological parameters. Codified information about the intake of the dietary supplement or placebo for both sides, patients and physicians as well, is the best way to avoid possible bias.

On the other hand, this RCT offers many advantages for patients recruited, including the lipoproteins profile for CVDs prevention and a complete check-up. The unbalance of the lipid profile is a complex phenomenon dependent on the interaction between lifestyle and genetic make-up. This study was planned to monitor physical activity through a dedicated questionnaire, to complete the beginning and the end of the study to avoid possible bias associated with lifestyle changes. Unifying biochemical, clinical, and data originating from medical history and physical activity questionnaire, this study gives a comprehensive picture of the lipid profile of the patients.

Even if the body weight will be recorded during the study, the weak point of this trial could be that a nutrition-dedicated questionnaire is missing. ESC/ESA guidelines contain a list of foods and nutraceutical compounds that has lipid-lowering effect being explored in randomized, controlled clinical trials [27]. Finally, this protocol study planned to verify the effects of BruMeChol™ dietary supplementation in subjects with mild hypercholesterolemia could also contribute to new study designs for next large-scale multicenter clinical trials. This kind of clinical trials is necessary to be done not only to study potential synergistic effects of a new mixture of natural compounds but also to investigate its safety.

### Trial status

Patient recruitment stage (study protocol version 3_20-Dec-2017). Patient recruitment has been started on 6 March 2018. The recruitment will be completed approximately on the end of the month of July 2020.

## Data Availability

Not applicable

## Abbreviations

CVDs: Cardiovascular diseases
Il: Interleukins
miRs-21: MicroRNAs-21
LDL: Low-density lipoprotein
*hs*-CRP: High-sensitive C-reactive protein
CV: Cardiovascular
CKD-EPI e GFR: Calculation of estimated glomerular filtration rate by CKD-EPI equation
AST/GOT: Aspartate aminotransferase glutamic oxaloacetic transaminase
ALT/GPT: Alanine aminotransferase/glutamic pyruvic transaminase
HDL: High-density cholesterol
WBCs: White blood cells
RBCs: Red blood cells
MCV: Mean cell volume
MCH: Mean corpuscular hemoglobin
MCHC: Mean corpuscular hemoglobin concentration
RDW: Red cell distribution width
CK: Creatine kinase
ECG: Electrocardiography
TSH: Thyroid-stimulating hormone
Lp(a): Lipoprotein(a)
CRF: Case report form
IPAQ-SF: International Physical Activity Questionnaire
CRP: C-reactive protein
GCP: Good clinical practice
